# Longitudinal changes in sputum and blood inflammatory mediators during FeNO suppression testing

**DOI:** 10.1136/thoraxjnl-2021-217994

**Published:** 2022-07-08

**Authors:** Simon Couillard, Rahul Shrimanker, Samuel Lemaire-Paquette, Gareth M Hynes, Catherine Borg, Clare Connolly, Samantha Jane Thulborn, Angela Moran, Sarah Poole, Sophie Morgan, Timothy Powell, Ian Pavord, Timothy Hinks

**Affiliations:** 1 Respiratory Medicine Unit, Nuffield Department of Medicine, University of Oxford, Oxford, UK; 2 Faculté de Médecine et des Sciences de la Santé, Université de Sherbrooke, Sherbrooke, Quebec, Canada

**Keywords:** Asthma, Pulmonary eosinophilia, Exhaled Airway Markers, Airway Epithelium, Asthma Mechanisms

## Abstract

To explore whether fractional exhaled nitric oxide (FeNO) non-suppression identifies corticosteroid resistance, we analysed inflammatory mediator changes during a FeNO suppression test with monitored high-intensity corticosteroid therapy. In linear mixed-effects models analysed over time, the 15 clinically distinct ‘suppressors’ (ie, *≥*42% FeNO suppression) normalised Asthma Control Questionnaire scores (mean±SD, start to end of test: 2.8±1.4 to 1.4±0.9, p<0.0001) and sputum eosinophil counts (median (IQR), start to end of test: 29% (6%–41%) to 1% (1%–5%), p=0.0003) while significantly decreasing sputum prostaglandin D_2_ (254 (89–894) to 93 (49–209) pg/mL, p=0.004) and numerically decreasing other type-2 cytokine, chemokine and alarmin levels. In comparison, the 19 non-suppressors had persistent sputum eosinophilia (10% (1%–67%) despite high-intensity therapy) with raised end-test inflammatory mediator levels (1.9 (0.9–2.8)-fold greater than suppressors). FeNO non-suppression during monitored treatment implies biological corticosteroid resistance.

## Introduction

Severe asthma represents 1 in 20 asthma cases but comprises half of asthma-related expenditure.^1^ The biomarkers fractional exhaled nitric oxide (FeNO) and blood eosinophils are used in the clinic to identify higher risk type 2 inflammatory phenotype which responds favourably to anti-inflammatory therapy.^2 3^

The observation that FeNO predicts inhaled corticosteroid (ICS)-responsiveness has led to the development of the FeNO suppression test to identify non-adherence in difficult-to-treat, FeNO-high asthma.^4 5^ One-third of patients have a persistently raised FeNO and disease burden despite objectively measured adherence to high-dose ICS.^4 6–8^ This group of ‘FeNO non-suppressors’ have been presumed ‘corticosteroid resistant’,^9^ but the longitudinal investigation of inflammatory changes over the course of a FeNO suppression test has not been reported. To explore the hypothesis that FeNO non-suppression identifies biological corticosteroid resistance, we analysed induced sputum and blood inflammatory mediator changes during a FeNO suppression test in patients who did and did not suppress FeNO.

## Methods

We performed an observational longitudinal analysis of FeNO suppression tests conducted in our specialist asthma clinic (Oxford, UK).

Patients ≥18 years old with asthma receiving high dosage ICS plus ≥1 other controller were recruited after multidisciplinary evaluation when they had persistently high FeNO (>40 ppb twice)^10^ with no confounding pulmonary disease. Participants consented and underwent testing between January 2015 and February 2020; sputum induction and recruitment stopped in March 2020 due to the pandemic.

FeNO suppression tests were conducted according to an adaptation of an early protocol (see Figure E2, online supplement).^4^ Briefly, patients with asthma underwent 7–35 days of additional inhaled and/or systemic corticosteroids (+1000 µg inhaled fluticasone propionate per day and, if FeNO did not suppress on day 7 according to the equation below, +80 mg intramuscular (IM) triamcinolone with follow-up 28 days later). Treatment adherence was monitored via a chipped inhaler (INCA) and/or nurse-administered triamcinolone injection. In addition to detailed clinical assessment, Asthma Control Questionnaire (ACQ)-5, spirometry, FeNO measurement (FeNO NIOX VERO), phlebotomy and sputum induction by hypertonic saline nebulisation in clinic on days 0, 7 and/or 35, patients performed daily FeNO measurements at home for days 1–6. Some FeNO suppression tests stopped after 7 days due to patient availability, physician decision or transition to research bronchoscopy protocols.

10.1136/thoraxjnl-2021-217994.supp1 Supplementary data

A positive FeNO suppression test was defined as a Log_10_∆FeNO ≥0.24, where Log_10_∆FeNO is calculated as: (mean (log_10_FeNO day 0, log_10_FeNO day 1)) − (log_10_FeNO day 35 or, if unavailable, mean (log_10_FeNO day 6, Log_10_FeNO day 7)). Conversely, patients with a negative FeNO suppression test (ie, <42% fall in FeNO) were categorised as ‘non-suppressors’.^4^ Medical notes and forms completed on day 0, 7 and 35 were reviewed to assess whether evidence of pre-existing nonadherence issues had been documented. Triggers for categorising patients as ‘previously non-adherent’ were any of: (1) adequate chipped inhaler data showing <70% acceptable doses taken during the first 7 days of the test, (2) ‘non-adherent’ noted during clinical review by specialist nurse or (3) nursing note stating significant inhaler technique difficulties persisting throughout the test.

Longitudinal (days 0, 7 and 35 whenever available) samples were analysed for 28 clinical, biomarker, sputum and serum inflammatory mediators. Inflammatory proteins were measured in duplicates using multiplex electrochemiluminescent assays (Meso Scale Discovery, USA) or single ELISA (Cayman Chemical, USA).

Demographics for FeNO suppressors versus non-suppressors were compared by unpaired t-tests for parametric variables, Mann-Whitney tests for nonparametric variables, and Fisher’s exact test or χ^2^ for categorical variables. To test our hypothesis that FeNO non-suppressors exhibit biological resistance, longitudinal analyses were performed for the 28 outcome repeated measures (days 0, 7 and 35 whenever data were available; plus home-FeNO measurement on days 1–6) in linear mixed-effects models with a random intercept on same patients for (1) FeNO non-suppressors alone and FeNO suppressors alone, respectively, assessing significance of change over timepoints in each subgroup; and (2) FeNO suppressors versus FeNO non-suppressors, assessing significance of the group **×** time interaction (ie, whether change over time was different according to group status). Significant findings in the longitudinal groupwise analyses were further explored in pooled linear mixed effects models assessing the relationship between selected continuous outcomes (ie, the dependent variable; log-transformed when required) and FeNO (independent variable; log-transformed). Modelling assumptions were all verified visually with appropriate diagnostic plots. The primary set of linear mixed-effect models’ p values (84 models) were controlled for a false discovery rate <0.05 using the Benjamini-Hochberg procedure^11^; other statistics used a two-sided α=0.05. Linear mixed-effects models were computed in RStudio 2021.09.01 build 372 (RStudio, USA) with R V.4.1.2 (R Foundation), and other statistics were performed in SPSS V.28 (IBM) and GraphPad Prism V.9.3.1 (GraphPad, USA).

## Results

Eighty-seven patients were referred for FeNO suppression testing between January 2015 and February 2020; 34 completed tests were included (see online supplemental appendix 1). There were two protocol deviations when FeNO non-suppressors were not administered IM triamcinolone on day 7 due to incorrect application of the FeNO suppression equation stated in the study methods (eg, using only 1 day to determine if suppressed, rather than the mean of several days).

Nineteen patients did not suppress FeNO: these were significantly older, on higher background ICS dosage, had lower baseline blood eosinophil count and had little or no adherence/inhaler technique issues noted (table 1). Specimen availability was low, especially for sputum differential cell counts, but there was no difference in the number of sputum inductions achieved between groups and no trend for better/worst sampling success according to study day.

**Table 1:** Baseline subject characteristics

Parameter	FeNO suppressed	Not suppressed	P value
	n=15	n=19	
Age, years	42±13	57±16	0.006
Male	5 (33)	10 (53)	ns
BMI, kg/m²	26±4	28±5	ns
Comorbidities			
Atopy*	12 (80)	12 (63)	ns
Nasal polyps	7 (47)	7 (37)	ns
Gastro-oesophageal reflux	2 (13)	3 (16)	ns
Cardiovascular disease	2 (13)	1 (5)	ns
Smoking status: never-smoker	12 (80)	11 (58)	ns
Ex-smoker	2 (13)	7 (37)	
Current smoker	1 (7)	1 (5)	
ACQ-5 score at baseline	2.8±1.4	2.5±1.5	ns
Asthma attacks in past year†	1 [0–3]	4 [0–5]	ns
ICS, BDP-CFC eq., μg/day	1561±502	1921±344	0.02
On maintenance OCS	3 (20)	9 (47)	ns
FEV_1_, % predicted	89±19	78±17	ns
FEV_1_/FVC ratio, % observed	75±17	67±11	ns
FeNO ppb	119 [75-190]	94 [60-136]	ns
Blood eosinophils, cells×10^9^ /L	0.54 [0.50–0.83]	0.46 [0.36–0.59]	0.03
Total IgE levels, kU/L	545 [35–1551]	229 [77-359]	ns
Sputum eosinophils, %	29 [7-41]	13 [3-39]	ns
Sputum neutrophils, %	46 [19-61]	68 [32-77]	ns
Inadequate adherence identified	8 (53)	2 (11)	0.007
Test duration			
7 days	5 (33)	11 (58)	ns
35 days	10 (67)	8 (42)	
Test optimisation method:			
+FP 1000 µg inhaled-only	12 (80)	13 (68)	ns
+FP then Triamcinolone 80 mg IM	3 (20)	6 (32)	
No of samples (days 0, 7, 35)			
Sputum differential cell count	21 {7, 10, 4}	17 {8, 7, 2}	
Sputum supernatant	25 {9, 9, 7}	31 {13, 12, 6}	
Serum	30 {11, 10, 9}	41 {17, 16, 8}	

Data are presented as no (%), mean±SD, median (IQR), or total no of samples (days 0, 7, 35).

P values reported are unpaired t-tests for parametric variables, Mann-Whitney U tests for nonparametric variables, Fisher’s exact test or χ^2^ for categorical variables.

*Atopy defined as patient-reported allergic rhinitis, eczema, allergen-worsening of asthma or food allergy.

†Asthma attacks are defined as acute asthma episodes requiring 3 days or more of systemic corticosteroids.

ACQ-5, Asthma Control Questionnaire-5 Item; BDP-CFC eq., beclomethasone dipropionate with CFC propellant equivalent; BMI, body mass index; FeNO, fractional exhaled nitric oxide; FEV1, forced expiratory volume in 1 s (postbronchodilator); FP, fluticasone propionate; FVC, forced vital capacity; ICS, inhaled corticosteroid; IM, intramuscular; ns, not significant; OCS, oral corticosteroids.

The clinical, biomarker and sputum/serum inflammatory longitudinal responses during the FeNO suppression tests are shown in table 2, and linear mixed-effect models’ outputs are detailed in online supplemental appendix 2. In FeNO suppressors alone, ACQ-5 scores improved significantly during the test (days 0, 7, 35; mean±SD: 2.8±1.4, 1.6±0.9, 1.3±1.0, p<0.0001 over time), as did sputum eosinophils (median (IQR): 29 (6–41), 3 (1–11), 2 (1–5) %, p=0.0003) and sputum PGD_2_ (254 (89–894), 174 (37–341), 93 (53–196) pg/mL, p=0.004). In FeNO non-suppressors alone, only the longitudinal change in sputum IL-4 (1.0 (0.3–1.1), 1.0 (0.5–1.2), 0.1 (0.1–0.3) pg/mL, p=0.004) was retained after correcting for multiplicity of testing. When comparing FeNO suppressors and non-suppressors, only the longitudinal change in FeNO was significantly different after correcting for multiplicity of testing (↓3.4 (2.3–4.2) vs ↓1.5 (1.1–1.7)-fold, p<0.0001 for group **×** time interaction).

**Table 2:** Before-and-after clinical and inflammatory changes according to FeNO suppression test result

Analyte (pg/mL or stated) LLOD*		FeNO suppressed			FeNO not suppressed			P for group ×time
		Before	After	P for time (n analysed)	Before	After	*P* for time (n analysed)	
**Clinical**	**ACQ-5 score**	2.8±1.4	1.4±0.9	**<0.0001** (n=15)	2.5±1.5	1.9±1.3	ns (n=19)	ns
	**FEV**_**1**_ **(L)**	2.79±0.86	3.05±0.96	0.009 (n=15)	2.39±0.92	2.56±0.89	0.04 (n=19)	ns
	**FEV**_**1**_ **(% pred)**	89±19	98±19	0.02 (n=15)	78±17	83±18	ns (n=19)	ns
	**FEV**_**1**_ **/FVC (%)**	75±17	78±12	ns (n=15)	67±11	70±10	ns (n=19)	ns
**Biomarker**	**FeNO (ppb)**	119 [75–190]	35 [20–55]	**<0.0001** (n=15)	94 [60–136]	56 [43–123]	**<0.0001** (n=19)	**<0.0001**
	**Blood Eos (×10** ^**9**^ ** /L)**	0.54 [0.50–0.83]	0.42 [0.10–0.60]	0.02 (n=15)	0.46 [0.26–0.58]	0.24 [0.19–0.36]	ns (n=19)	ns
**Induced sputum mediators**	**Eosinophils** **(%)**	29.3 [6.5–41.3]	1.3 [1.0–5.3]	**0.0003** (n=11)	13.0 [2.9–38.8]	10.0 [1.1–67.0]	ns (n=10)	ns
	**Neutrophils** **(%)**	46.3 [9.8–61.3]	16.0 [4.7–74.7]	ns (n=11)	67.8 [32.0–77.3]	40.3 [8.5–70.3]	ns (n=10)	ns
	**IL-4** 0.2	0.4 [0.1–1.0]	0.1 [0.1–0.6]	ns (n=11)	1.0 [0.3–1.1]	0.5 [0.1–1.0]	**0.002** (n=13)	ns
	**IL-5** 0.5	3.7 [1.2–20.9]	1.4 [0.6–6.0]	0.045 (n=11)	7.8 [1.9–14.5]	3.9 [2.0–7.4]	ns (n=13)	ns
	**IL-13** 4.2	6.9 [5.7–15.8]	8.8 [6.0–15.5]	ns (n=11)	7.7 [5.4–10.5]	7.7 [6.3–10.8]	ns (n=13)	ns
	**IL-33** 0.6	1.4 [0.3–1.4]	0.3 [0.3–0.7]	0.02 (n=11)	1.6 [1.4–2.0]	1.4 [0.4–1.7]	0.02 (n=13)	ns
	**TSLP** 0.9	3.6 [1.3–13.9]	3.0 [1.1–7.9]	0.008 (n=11)	7.0 [5.0–13.4]	6.9 [4.1–10.3]	ns (n=13)	ns
	**Eotaxin-3** 4.2	63 [24–410]	58 [14–257]	ns (n=9)	361 [20–677]	169 [48–329]	ns (n=11)	ns
	**TARC** 0.4	10 [7–79]	16 [5–42]	ns (n=9)	36 [8–208]	31 [17–48]	ns (n=11)	ns
	**LTE**_**4**_ 7.8	305 [74–830]	106 [46–218]	0.01 (n=11)	226 [54–905]	80 [47–677]	ns (n=13)	ns
	**PGD**_**2**_ 19.5	254 [89–894]	93 [49–209]	**0.004** (n=11)	279 [151–366]	176 [119–320]	0.04 (n=13)	0.01
	**IFN-γ** 0.3	0.6 [0.2–1.7]	0.2 [0.2–0.3]	ns (n=9)	0.2 [0.2–0.4]	0.4 [0.2–1.1]	ns (n=11)	ns
	**TNF** 0.4	1.8 [0.2–9.8]	0.5 [0.2–2.4]	ns (n=9)	1.7 [0.9–4.0]	1.7 [0.2–7.3]	ns (n=11)	ns
**Serum mediators**	**IL-4** 0.1	0.1 [0.1–0.1]	0.1 [0.1–0.1]	ns (n=14)	0.1 [0.1–0.1]	0.1 [0.1–0.1]	ns (n=19)	ns
	**IL-5** 0.4	1.4 [0.6–3.4]	0.8 [0.5–1.2]	ns (n=14)	1.5 [0.4–2.4]	0.6 [0.5–1.6]	ns (n=19)	ns
	**IL-13** 6.7	9.5 [3.3–12.0]	3.3 [3.3–8.5]	ns (n=14)	3.3 [3.3–12.8]	6.1 [3.3–10.5]	ns (n=19)	ns
	**IL-33** 0.4	0.8 [0.2–0.8]	0.2 [0.2–0.8]	ns (n=14)	0.6 [0.2–0.8]	0.4 [0.2–0.8]	ns (n=19)	ns
	**TSLP** 0.5	1.8 [0.8–3.1]	1.8 [1.1–2.5]	ns (n=14)	2.7 [1.8–3.6]	2.4 [1.6–3.8]	ns (n=19)	ns
	**Eotaxin-3** 4.2	14 [6–30]	15 [10–30]	ns (n=14)	19 [10–35]	17 [9–32]	0.03 (n=19)	ns
	**TARC** 0.2	281 [167–561]	318 [160–560]	0.01 (n=14)	247 [144–395]	248 [92–406]	ns (n=19)	ns
	**IFN-γ** 0.3	0.6 [0.2–1.1]	0.4 [0.3–0.7]	ns (n=14)	0.3 [0.2–1.0]	0.3 [0.2–0.8]	ns (n=19)	ns
	**TNF** 0.4	0.8 [0.2–1.3]	1.0 [0.5–1.9]	ns (n=14)	1.1 [0.2–2.1]	0.9 [0.2–2.0]	ns (n=19)	ns

Data are presented as mean±SD or median (IQR); units of measured are in pg/mL unless otherwise stated.

Bold p-values are those retained after controlling for multiplicity of testing (false discovery threshold 0.05 across 84 analyses). P values reported were obtained by linear mixed effects models.

*Cytokine levels that were not quantified were assigned the arbitrary value of 0.5×the LLOD (value below the row label when appropriate) to allow analysis.

ACQ-5, 5-item Asthma Control Questionnaire; Eos, eosinophils; FeNO, fractional exhaled nitric oxide; FEV1, forced expiratory volume in 1 s s (postbronchodilator); FVC, forced vital capacity; IFN, interferon; IL, interleukin; LLOD, lower limit of detection; LTE4, leukotriene E4; ns, not significant; PGD2, prostaglandin D2; TARC, thymus activation regulated cytokine (CCL17); TNF, tumour necrosis factor; TSLP, thymic stromal lymphopoietin.

The results of the above subgroup longitudinal analyses were further explored for ACQ-5, sputum eosinophils, sputum PGD_2_ and sputum IL-4. The continuous relationship between FeNO suppression and these analytes are detailed in online supplemental appendix 3. In effect, a 42% decrease in FeNO is associated with a significant change in ACQ-5 (↓0.31 (95% CIs: 0.20 to 0.42) points, p<0.0001), sputum eosinophils (↓ 1.37 (1.10 to 1.72)-fold, p=0.009) and sputum PGD_2_ (↓ 1.16 (1.01 to 1.32)-fold, p=0.04). There was no significant relationship between the degree of FeNO suppression and sputum IL-4.

**Figure 1:**
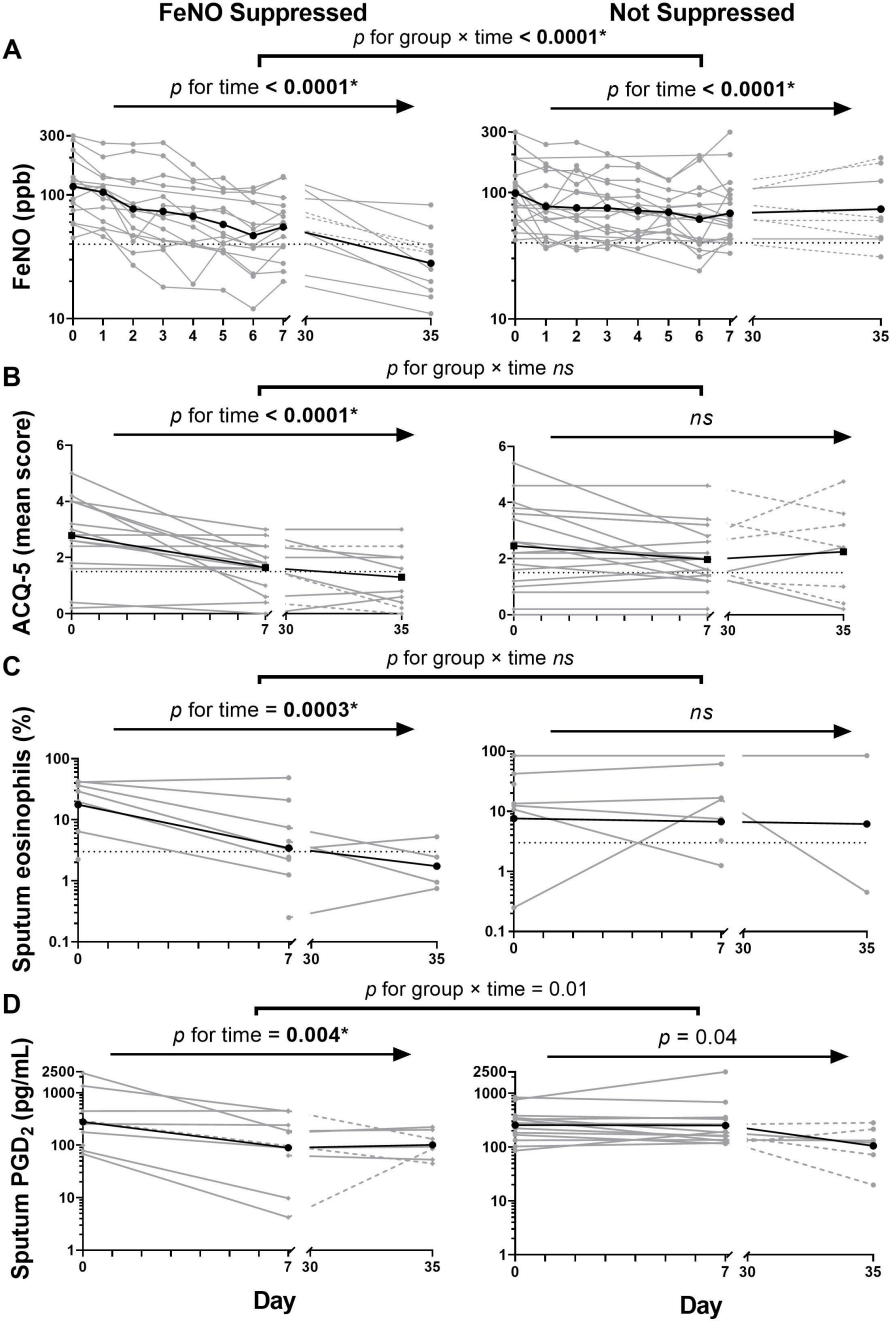
Longitudinal changes in selected analytes during a fractional exhaled nitric oxide (FeNO) suppression test stratified by its results. (A) FeNO (individual and geometric mean values), (B): 5-item Asthma Control Questionnaire (ACQ-5) (individual and mean values); (C): sputum eosinophils (individual and geometric mean values); (D): sputum prostaglandin D_2_ (PGD_2_) (individual and geometric mean values). Bold *p values are those retained after controlling for a false discovery rate <0.05; dashed segments (^_ _ _^) indicate patients administered IM triamcinolone on day 7; dotted horizontal lines (^…^) delineate the limits of normal/controlled asthma for FeNO (<40 ppb), ACQ-5 (<1.5) and sputum eosinophils (<3%).^10^ ns, not significant.

The four analytes found to significantly change in both subgroup and pooled continuous analyses according to FeNO suppression (FeNO, ACQ-5, sputum eosinophils and PGD_2_) are plotted (figure 1).

It is noteworthy that more outcome measures decreased with p<0.05 in FeNO suppressors than non-suppressors (11/28 vs 6/28, p=0.14 on χ^2^ test), and in nearly all cases the end-test median values for sputum and serum inflammatory mediators were numerically greater in FeNO non-suppressors than suppressors (1.9 (0.9–2.8)-fold; 15/22 values greater in FeNO non-suppressors, p=0.02 on χ^2^ test). Patients who did not suppress FeNO also had significantly greater FeNO values at test termination than suppressors (56 (43–123) vs 35 (20–55) ppb, unpaired t-test on log-FeNO values p=0.001). These trends were especially striking for sputum eosinophils (figure 1C), which decreased 5.4 (2.4–10.3)-fold in FeNO suppressors (~normal median end-test value: 1 (1–5) %, n=7) while increasing 1.3 (0.6–1.6)-fold in non-suppressors (~high median end-test value: 10 (1–67) %, n=6).

Finally, sensitivity analyses were conducted to assess whether the final degree of FeNO suppression or ACQ-5 improvement varied according to study duration (7 days or 35 days) and the optimisation method (ICS-only or ICS+IM triamcinolone) (online supplemental appendix 4). The results suggest that, although methods to ensure optimal FeNO suppression varied, the magnitude of change did not differ significantly between study durations and interventions.

## Discussion

We found that patients who failed to suppress FeNO after a suppression test had no improvement in symptoms and FeNO, reflected by raised sputum eosinophil counts, sputum PGD_2_, and other inflammatory protein levels at the end of the test. In contrast, FeNO suppressors improve significantly in these domains, often reaching normal values. These results suggest that the assessment of biological corticosteroid resistance can be based on a failure to suppress FeNO during monitored high-intensity corticosteroid therapy.

The criterion for FeNO suppression was derived to identify pre-existing nonadherence—not to assess corticosteroid-resistant type-2 inflammation.^4^ Nevertheless, patients who failed to suppress FeNO have consistently been found to be older males with higher baseline asthma morbidity and lesser longitudinal improvements in symptom scores, lung function, and FeNO.^4 6–8^ Our data confirm these distinct clinical characteristics and provide translational data supporting the concept that FeNO non-suppression identifies corticosteroid resistance.^9^ They also highlight how monitoring adherence allows better interpretation of FeNO fluctuations.^12^ An important strength of our study is that we rigorously controlled for multiplicity of testing. Furthermore, we validated the significant findings from longitudinal subgroup analyses (FeNO suppressed, not suppressed) by modelling them according to the degree suppression of FeNO. Hence, FeNO suppression (taken both as a categorical and a continuous variable) translates to a normalisation of the ACQ-5 score, sputum eosinophil count and sputum PGD_2_; a mast cell-produced eicosanoid with proinflammatory and bronchoconstrictive effects.^13^ Conversely, the clinically distinct FeNO non-suppressors have corticosteroid-refractory symptoms and airway inflammation.

Notwithstanding the results of our subgroup longitudinal analyses which confirmed our study hypothesis, we were unable to show a comparative difference between the two groups across time, possibly because the assessment of the group×time statistical interaction was underpowered to detect the likely difference. Sputum availability in our cohort was also problematic and the study was thus generally underpowered despite robust linear mixed-effect modelling efforts to use all the data at hand. Reports on sputum induction success rates reach 92%^14^; our rate was 44% for differential cell counts and 65% for sputum supernatants. Serum samples were more available (83%) but less useful to assess FeNO-related mechanisms. Another limitation of this study is its observational design with consequent heterogenous testing durations and interventions, although sensitivity analyses did not show any significant impact of these factors on FeNO and symptom improvements. Despite these limitations, the number of inflammatory mediator changes in contrasting directions between suppressors and non-suppressors were unlikely to be just stochastic.

To conclude, our longitudinal subgroup support the notion that patients with uncontrolled asthma who fail to suppress FeNO despite monitored high-intensity corticosteroid therapy have distinct clinical, biomarker and inflammatory mediator responses which imply biological corticosteroid resistance. Further comparative biological analyses between FENO suppressors and non-suppressors require larger validation cohorts and sample sets.
