# Supplementary material for: Longitudinal changes in sputum and blood inflammatory mediators during FeNO suppression testing
Source: Thorax. 2022 Jul 8;77(9):933–8. doi: 10.1136/thoraxjnl-2021-217994 (PMC9411876; doi:10.1136/thoraxjnl-2021-217994)
Supplement: Supplementary data [file thoraxjnl-2021-217994supp001.pdf]

Longitudinal changes in sputum and blood inflammatory mediators during FeNO suppression testing

Simon Couillard, Rahul Shrimanker, Samuel Lemaire-Paquette, Gareth M Hynes, Catherine Borg, Clare Connolly, Samanatha Thulborn, Angela M Moran, Sarah Poole, Sophie Morgan, Timothy Powell, Ian D Pavord, Timothy S C Hinks.

Supplementary Material

Couillard S, *et al. Thorax* 2022; 77:933–938. doi: 10.1136/thoraxjnl-2021-217994

Table of Contents

**Content**	**Page**
**Appendix 1** Flowchart of patient enrolment process	E3
**Appendix 2** Detailed outputs from linear mixed effects modelling	E4
**Appendix 3** Results of pooled linear mixed effect models exploring the continuous relationships between FeNO and key analytes	E10
**Appendix 4** Sensitivity analyses for different test durations and optimisation methods	E11

APPENDIX 1 – Flowchart of patient enrolment process

Eighty-seven FeNO suppression tests were planned between 2015 and 2020; 34 completed tests were retained after applying inclusion/exclusion criteria (Figure E1).

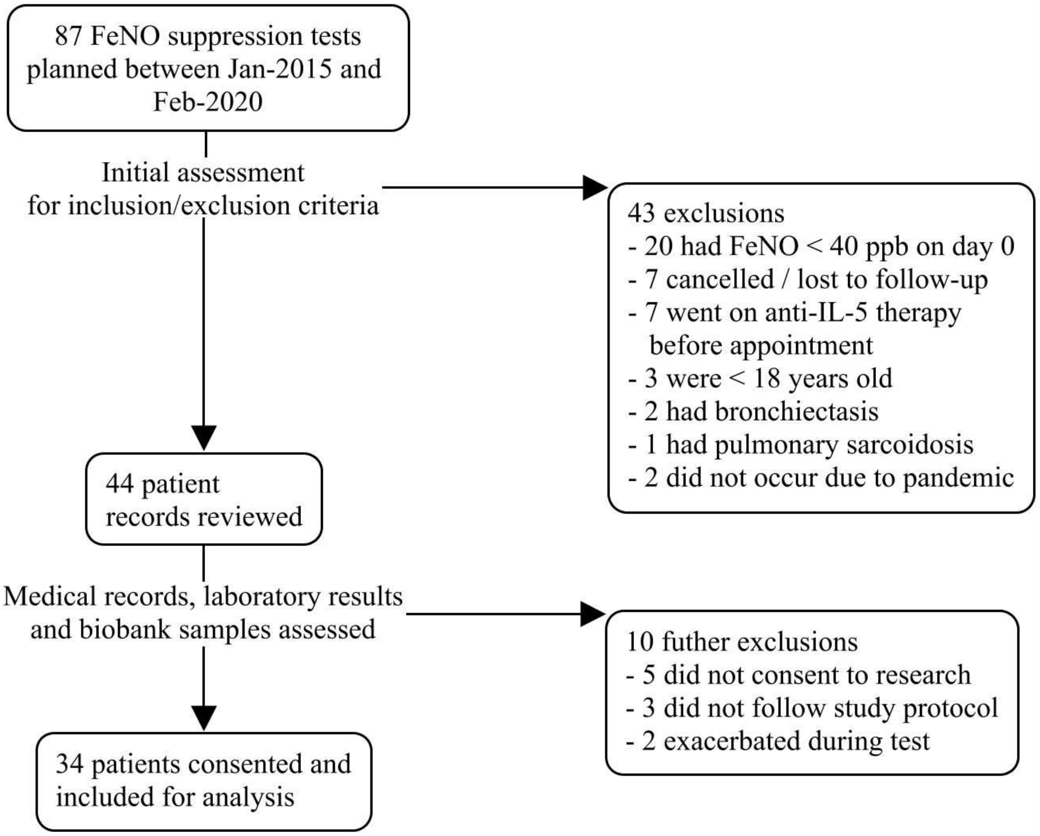

FIGURE E1. Flowchart for fractional exhaled nitric oxide (FeNO) suppression study inclusion. IL-5, interleukin 5.

APPENDIX 2 – Detailed outputs from linear mixed effects modelling

TABLE E1
Linear mixed effect model outputs within groups to assess suppression over time

	Analyte	Variable	FeNO suppressed (N=15) Coeff.	Lower 95%CI	Upper 95%CI	P-value (n)	FeNO not suppressed (N=19) Coeff.	Lower 95%CI	Upper 95%CI	P-value (n)
**Clincal**	**ACQ-5 score**	Time 2 (Day 7)	-1.15	-1.71	-0.58	2.8E-06	-0.48	-0.97	0.00	0.08
		Time 3 (Day 35)	-1.50	-2.15	-0.85	(n=15)	-0.61	-1.29	0.07	(n=19)
	**FEV_1, L**	Time 2 (Day 7)	0.16	0.01	0.31	0.009	0.05	-0.12	0.22	0.04
		Time 3 (Day 35)	0.26	0.09	0.44	(n=15)	0.31	0.07	0.55	(n=19)
	**FEV_1, % pred**	Time 2 (Day 7)	6.15	0.20	12.11	0.02	1.81	-3.98	7.60	0.06
		Time 3 (Day 35)	9.62	2.66	16.57	(n=15)	9.75	1.59	17.91	(n=19)
	**FEV_1/FVC, %**	Time 2 (Day 7)	2.01	-1.79	5.81	0.4	0.06	-3.48	3.61	0.5
		Time 3 (Day 35)	2.82	-1.61	7.26	(n=15)	2.73	-2.25	7.72	(n=19)
**Biomark**	**Log_e(FeNO, ppb)**	Time 2 (Day 7)	0.47	0.37	0.60	**3.9E-07**	0.69	0.61	0.80	**2.2E-16**
		Time 3 (Day 35)	0.23	0.17	0.31	(n=15)	0.72	0.59	0.88	(n=19)
	**Log_e(Blood Eos, ×10^9/L)**	Time 2 (Day 7)	0.62	0.41	0.94	0.02	0.73	0.49	1.09	0.09
		Time 3 (Day 35)	0.57	0.37	0.88	(n=15)	0.61	0.39	0.97	(n=19)
**Induced sputum mediators**	**Log_e(Eosinophils, %)**	Time 2 (Day 7)	0.24	0.11	0.53	**0.0003**	1.18	0.23	6.11	0.9
		Time 3 (Day 35)	0.15	0.05	0.43	(n=11)	0.71	0.05	9.24	(n=10)
	**Log_e(Neutrophils, %)**	Time 2 (Day 7))	1.08	0.56	2.06	0.9	0.23	0.06	0.97	0.1
		Time 3 (Day 35)	1.21	0.50	2.94	(n=11)	0.71	0.08	6.46	(n=10)
	**Log_e(IL-4, pg/mL)**	Time 2 (Day 7)	0.63	0.36	1.10	0.1	1.18	0.73	1.89	**0.002**
		Time 3 (Day 35)	0.54	0.29	1.01	(n=11)	0.39	0.21	0.71	(n=13)
	**Log_e(IL-5, pg/mL)**	Time 2 (Day 7)	0.29	0.11	0.78	0.045	0.69	0.29	1.66	0.6
		Time 3 (Day 35)	0.42	0.14	1.25	(n=11)	0.60	0.20	1.82	(n=13)
	**Log_e(IL-13, pg/mL)**	Time 2 (Day 7)	0.86	0.63	1.19	0.5	1.00	0.71	1.42	1.0
		Time 3 (Day 35)	1.03	0.72	1.46	(n=11)	1.01	0.65	1.56	(n=13)
	**Log_e(IL-33, pg/mL)**	Time 2 (Day 7)	0.60	0.33	1.09	0.02	0.93	0.55	1.58	0.02
		Time 3 (Day 35)	0.40	0.21	0.76	(n=11)	0.41	0.21	0.77	(n=13)
	**Log_e(TSLP, pg/mL)**	Time 2 (Day 7)	0.52	0.34	0.80	0.008	0.80	0.42	1.53	0.4
		Time 3 (Day 35)	0.82	0.51	1.31	(n=11)	0.60	0.27	1.31	(n=13)
	**Log_e(Eotaxin-3, pg/mL)**	Time 2 (Day 7)	0.27	0.05	1.51	0.3	1.69	0.25	11.28	0.6
		Time 3 (Day 35)	0.36	0.07	1.85	(n=9)	0.62	0.10	3.77	(n=11)

(Table E2 continued)

	Analyte	Variable	FeNO suppressed (N=15) Coeff.	Lower 95%CI	Upper 95%CI	P-value (n)	FeNO not suppressed (N=19) Coeff.	Lower 95%CI	Upper 95%CI	P-value (n)
**Sputum mediators**	**Log$_e$(TARC, pg/mL)**	Time 2 (Day 7)	0.48	0.19	1.21	0.2	0.74	0.27	2.02	0.5
		Time 3 (Day 35)	0.48	0.20	1.14	(n=9)	0.59	0.22	1.56	(n=11)
	**Log$_e$(LTE$_4$, pg/mL)**	Time 2 (Day 7)	0.28	0.12	0.65	0.01	0.72	0.36	1.46	0.5
		Time 3 (Day 35)	0.40	0.16	1.01	(n=11)	0.61	0.24	1.55	(n=13)
	**Log$_e$(PGD$_2$, pg/mL)**	Time 2 (Day 7)	0.28	0.13	0.62	**0.004**	0.99	0.63	1.55	0.04
		Time 3 (Day 35)	0.34	0.14	0.81	(n=11)	0.49	0.28	0.89	(n=13)
	**Log$_e$(IFN-γ, pg/mL)**	Time 2 (Day 7)	0.40	0.12	1.33	0.3	1.75	0.80	3.85	0.3
		Time 3 (Day 35)	0.53	0.17	1.66	(n=9)	1.50	0.69	3.25	(n=11)
	**Log$_e$(TNF, pg/mL)**	Time 2 (Day 7)	0.76	0.25	2.30	0.4	0.57	0.18	1.76	0.5
		Time 3 (Day 35)	0.49	0.17	1.38	(n=9)	0.55	0.18	1.66	(n=11)
**Serum**	**Log$_e$(IL-4, pg/mL)**	Time 2 (Day 7)	0.68	0.43	1.07	0.2	(incalculable as all values equal)			1.0
		Time 3 (Day 35)	0.74	0.46	1.18	(n=14)				(n=19)
	**Log$_e$(IL-5, pg/mL)**	Time 2 (Day 7)	0.63	0.35	1.14	0.08	0.75	0.49	1.13	0.2
		Time 3 (Day 35)	0.51	0.28	0.94	(n=14)	0.64	0.37	1.09	(n=19)
	**Log$_e$(IL-13, pg/mL)**	Time 2 (Day 7)	0.84	0.65	1.09	0.1	1.02	0.91	1.15	0.6
		Time 3 (Day 35)	0.73	0.54	0.98	(n=14)	0.94	0.81	1.10	(n=19)
	**Log$_e$(IL-33, pg/mL)**	Time 2 (Day 7)	0.86	0.75	0.99	0.1	0.90	0.78	1.04	0.3
		Time 3 (Day 35)	0.91	0.78	1.07	(n=14)	0.90	0.75	1.10	(n=19)
	**Log$_e$(TSLP, pg/mL)**	Time 2 (Day 7)	0.88	0.57	1.35	0.5	0.89	0.59	1.34	0.06
		Time 3 (Day 35)	0.74	0.46	1.21	(n=14)	0.53	0.31	0.89	(n=19)
	**Log$_e$(Eotaxin-3, pg/mL)**	Time 2 (Day 7)	1.20	0.87	1.66	0.07	0.76	0.52	1.11	0.03
		Time 3 (Day 35)	0.79	0.55	1.14	(n=14)	0.52	0.32	0.86	(n=19)
	**Log$_e$(TARC, pg/mL)**	Time 2 (Day 7)	0.84	0.68	1.03	0.007	0.87	0.18	4.22	0.9
		Time 3 (Day 35)	0.68	0.54	0.86	(n=14)	1.21	0.17	8.54	(n=19)
	**Log$_e$(IFN-γ, pg/mL)**	Time 2 (Day 7)	0.62	0.37	1.03	0.09	1.06	0.60	1.88	0.9
		Time 3 (Day 35)	1.04	0.59	1.82	(n=14)	1.15	0.56	2.39	(n=19)
	**Log$_e$(TNF, pg/mL)**	Time 2 (Day 7)	0.94	0.65	1.37	0.6	0.84	0.54	1.31	0.6
		Time 3 (Day 35)	0.81	0.53	1.24	(n=14)	1.10	0.61	1.97	(n=19)

Values and 95% confidence intervals (CI) were obtained by linear mixed-effects models with a random intercept on same patients. **Bold** *P*-values are those retained after controlling for multiplicity of testing: a false discovery threshold 0.05 was applied across 84 analyses including Group × Time p-values indicated in table E2. ACQ-5, 5-item asthma control questionnaire; Eos, eosinophils; FeNO, fractional exhaled nitric oxide; FEV_1, forced expiratory volume in 1 second (post-bronchodilator); FVC, forced vital capacity; IFN, interferon; IL, interleukin; LTE_4, leukotriene E_4; PGD_2, prostaglandin D_2; TNF, tumour necrosis factor; TARC, thymus activation regulated cytokine (CCL17); TSLP, thymic stromal lymphopoietin.

Couillard S, *et al. Thorax* 2022; 77:933–938. doi: 10.1136/thoraxjnl-2021-217994

TABLE E2
Linear mixed effect model outputs in the whole sample for Group × Time interaction

	Analyte (n included)	Variable	Coefficient	Lower 95%CI	Upper 95%CI	P-value
Clinical	**ACQ-5 score (n=34)**	Group	0.33	-0.52	1.19	0.4
		Time 2 (Day 7)	-0.48	-0.98	0.01	0.09
		Time 3 (Day 35)	-0.58	-1.27	0.11	
		Group × Time 2	-0.66	-1.41	0.08	0.09
		Group × Time 3	-0.92	-1.86	0.03	
	**FEV_1, L (n=34)**	Group	0.40	-0.23	1.04	0.2
		Time 2 (Day 7)	0.05	-0.10	0.20	0.02
		Time 3 (Day 35)	0.31	0.10	0.53	
		Group × Time 2	0.11	-0.12	0.34	0.5
		Group × Time 3	-0.05	-0.35	0.24	
	**FEV_1, % pred (n=34)**	Group	10.78	-2.37	23.94	0.1
		Time 2 (Day 7)	1.81	-3.75	7.36	0.046
		Time 3 (Day 35)	9.88	2.04	17.72	
		Group × Time 2	4.35	-4.02	12.71	0.5
		Group × Time 3	-0.35	-11.06	10.36	
	**FEV_1/FVC, % (n=34)**	Group	7.31	-1.07	15.69	0.09
		Time 2 (Day 7)	0.06	-3.39	3.52	0.5
		Time 3 (Day 35)	2.89	-2.00	7.77	
		Group × Time 2	1.95	-3.26	7.15	0.7
		Group × Time 3	-0.13	-6.80	6.54	
Biomarker	**Log_e(FeNO, ppb) (n=34)**	Group	1.18	0.80	1.76	0.4
		Time 2 (Day 7)	0.69	0.58	0.83	0.0002
		Time 3 (Day 35)	0.72	0.56	0.93	
		Group × Time 2	0.68	0.52	0.89	**3.0E-07**
		Group × Time 3	0.32	0.22	0.45	
	**Log_e(Blood Eos, $\times10^9$/L) (n=34)**	Group	1.44	0.89	2.35	0.1
		Time 2 (Day 7)	0.73	0.49	1.08	0.08
		Time 3 (Day 35)	0.61	0.39	0.96	
		Group × Time 2	0.85	0.48	1.52	0.9
		Group × Time 3	0.93	0.50	1.75	
Sputum mediators	**Log_e(Eosinophils, %) (n=21)**	Group	2.10	0.41	10.80	0.4
		Time 2 (Day 7)	1.25	0.36	4.30	0.8
		Time 3 (Day 35)	0.67	0.10	4.59	
		Group × Time 2	0.19	0.03	1.03	0.1
		Group × Time 3	0.21	0.02	2.55	
	**Log_e(Neutrophils, %) (n=21)**	Group	0.41	0.09	1.92	0.3
		Time 2 (Day 7)	0.22	0.07	0.72	0.04
		Time 3 (Day 35)	0.84	0.13	5.38	
		Group × Time 2	3.52	0.69	17.86	0.3
		Group × Time 3	1.04	0.10	11.18	

(Table E2 continued)

	Analyte (n analysed) Variable	Coefficient	Lower 95%CI	Upper 95%CI	P-value
**Sputum mediators**	**Log$_e$(IL-4, pg/mL) (n=24)** Group	0.55	0.25	1.21	0.1
	Time 2 (Day 7)	1.17	0.73	1.88	0.003
	Time 3 (Day 35)	0.40	0.22	0.73	
	Group × Time 2	0.54	0.26	1.13	0.09
	Group × Time 3	1.33	0.56	3.16	
	**Log$_e$(IL-5, pg/mL) (n=24)** Group	0.58	0.17	1.97	0.4
	Time 2 (Day 7)	0.69	0.29	1.62	0.6
	Time 3 (Day 35)	0.61	0.21	1.79	
	Group × Time 2	0.42	0.11	1.59	0.4
	Group × Time 3	0.69	0.15	3.24	
	**Log$_e$(IL-13, pg/mL) (n=24)** Group	1.18	0.71	1.95	0.5
	Time 2 (Day 7)	0.99	0.73	1.35	0.99
	Time 3 (Day 35)	1.02	0.68	1.51	
	Group × Time 2	0.87	0.54	1.42	0.8
	Group × Time 3	1.03	0.58	1.82	
	**Log$_e$(IL-33, pg/mL) (n=24)** Group	0.66	0.37	1.21	0.2
	Time 2 (Day 7)	0.92	0.55	1.54	0.03
	Time 3 (Day 35)	0.43	0.22	0.81	
	Group × Time 2	0.66	0.30	1.47	0.6
	Group × Time 3	0.91	0.37	2.28	
	**Log$_e$(TSLP, pg/mL) (n=24)** Group	0.49	0.21	1.17	0.1
	Time 2 (Day 7)	0.82	0.43	1.54	0.4
	Time 3 (Day 35)	0.56	0.25	1.25	
	Group × Time 2	0.71	0.27	1.90	0.6
	Group × Time 3	1.33	0.42	4.18	
	**Log$_e$(Eotaxin-3, pg/mL) (n=20)** Group	0.84	0.12	5.78	0.9
	Time 2 (Day 7)	1.42	0.23	8.57	0.7
	Time 3 (Day 35)	0.56	0.10	3.18	
	Group × Time 2	0.20	0.02	2.44	0.4
	Group × Time 3	0.64	0.06	6.99	
	**Log$_e$(TARC, pg/mL) (n=20)** Group	0.47	0.10	2.20	0.3
	Time 2 (Day 7)	0.73	0.27	1.95	0.5
	Time 3 (Day 35)	0.59	0.22	1.56	
	Group × Time 2	0.67	0.17	2.58	0.8
	Group × Time 3	0.81	0.22	2.97	
	**Log$_e$(LTE$_4$, pg/mL) (n=24)** Group	1.04	0.28	3.82	0.96
	Time 2 (Day 7)	0.72	0.36	1.44	0.5
	Time 3 (Day 35)	0.61	0.25	1.53	
	Group × Time 2	0.39	0.13	1.18	0.2
	Group × Time 3	0.65	0.18	2.42	

(Table E2 continued)

Couillard S, *et al. Thorax* 2022; 77:933–938. doi: 10.1136/thoraxjnl-2021-217994

	Analyte (n analysed) / Variable	Coefficient	Lower 95%CI	Upper 95%CI	P-value
**Sputum mediators**	**Log$_e$(PGD$_2$, pg/mL) (n=24)** Group	1.11	0.46	2.67	0.8
	Time 2 (Day 7)	0.99	0.58	1.70	0.1
	Time 3 (Day 35)	0.50	0.25	1.01	
	Group × Time 2	0.29	0.12	0.67	0.01
	Group × Time 3	0.68	0.25	1.88	
	**Log$_e$(IFN-γ, pg/mL) (n=20)** Group	2.34	0.70	7.75	0.2
	Time 2 (Day 7)	1.93	0.60	6.14	0.4
	Time 3 (Day 35)	1.90	0.63	5.78	
	Group × Time 2	0.20	0.04	1.02	0.1
	Group × Time 3	0.28	0.06	1.30	
	**Log$_e$(TNF, pg/mL) (n=20)** Group	0.63	0.14	2.90	0.6
	Time 2 (Day 7)	0.56	0.18	1.77	0.5
	Time 3 (Day 35)	0.55	0.18	1.69	
	Group × Time 2	1.35	0.28	6.65	0.9
	Group × Time 3	0.87	0.19	4.01	
**Serum mediators**	**Log$_e$(IL-4, pg/mL) (n=23)** Group	1.49	1.16	1.93	0.002
	Time 2 (Day 7)	1.00	0.80	1.26	1.000
	Time 3 (Day 35)	1.00	0.75	1.32	
	Group × Time 2	0.68	0.47	0.98	0.09
	Group × Time 3	0.74	0.49	1.11	
	**Log$_e$(IL-5, pg/mL) (n=23)** Group	1.37	0.73	2.57	0.3
	Time 2 (Day 7)	0.77	0.49	1.21	0.4
	Time 3 (Day 35)	0.69	0.39	1.23	
	Group × Time 2	0.78	0.38	1.60	0.7
	Group × Time 3	0.70	0.30	1.61	
	**Log$_e$(IL-13, pg/mL) (n=23)** Group	1.07	0.65	1.75	0.8
	Time 2 (Day 7)	1.02	0.87	1.20	0.8
	Time 3 (Day 35)	0.95	0.77	1.18	
	Group × Time 2	0.83	0.64	1.08	0.2
	Group × Time 3	0.78	0.57	1.07	
	**Log$_e$(IL-33, pg/mL) (n=23)** Group	0.89	0.55	1.43	0.6
	Time 2 (Day 7)	0.90	0.79	1.03	0.3
	Time 3 (Day 35)	0.90	0.76	1.08	
	Group × Time 2	0.95	0.77	1.18	0.9
	Group × Time 3	1.00	0.77	1.30	
	**Log$_e$(TSLP, pg/mL) (n=23)** Group	0.54	0.30	0.99	0.046
	Time 2 (Day 7)	0.87	0.58	1.31	0.052
	Time 3 (Day 35)	0.52	0.31	0.88	
	Group × Time 2	1.13	0.59	2.16	0.4
	Group × Time 3	1.67	0.78	3.58	

(Table E2 continued)

Couillard S, *et al. Thorax* 2022; 77:933–938. doi: 10.1136/thoraxjnl-2021-217994

	Analyte (n anlaysed)	Variable	Coefficient	Lower 95%CI	Upper 95%CI	P-value
**Serum mediators**	**Log_e(Eotaxin-3, pg/mL) (n=23)**	Group	0.67	0.32	1.40	0.3
		Time 2 (Day 7)	0.76	0.54	1.06	0.01
		Time 3 (Day 35)	0.52	0.34	0.81	
		Group × Time 2	1.58	0.92	2.72	0.2
		Group × Time 3	1.51	0.79	2.90	
	**Log_e(TARC, pg/mL) (n=23)**	Group	2.85	0.70	11.53	0.1
		Time 2 (Day 7)	0.87	0.25	3.05	0.9
		Time 3 (Day 35)	1.21	0.26	5.72	
		Group × Time 2	1.07	0.14	8.10	0.99
		Group × Time 3	0.90	0.09	8.46	
	**Log_e(IFN-γ, pg/mL) (n=23)**	Group	1.09	0.53	2.24	0.8
		Time 2 (Day 7)	1.07	0.64	1.78	0.9
		Time 3 (Day 35)	1.16	0.60	2.24	
		Group × Time 2	0.59	0.26	1.35	0.4
		Group × Time 3	0.91	0.35	2.36	
	**Log_e(TNF, pg/mL) (n=23)**	Group	0.94	0.36	2.47	0.9
		Time 2 (Day 7)	0.84	0.56	1.24	0.5
		Time 3 (Day 35)	1.10	0.66	1.83	
		Group × Time 2	1.14	0.61	2.14	0.6
		Group × Time 3	0.76	0.35	1.62	

Values and 95% confidence intervals (CI) were obtained by linear mixed-effects models with a random intercept on same patients. The P-values surrounded by a solid square were included in the control for multiplicity of testing: a false discovery threshold 0.05 was applied across 84 analyses including those of table E1. **Bold** *P*-values are those retained after controlling for multiplicity of testing. ACQ-5, 5-item asthma control questionnaire; Eos, eosinophils; FeNO, fractional exhaled nitric oxide; FEV_1, forced expiratory volume in 1 second (post-bronchodilator); FVC, forced vital capacity; IFN, interferon; IL, interleukin; LTE_4, leukotriene E_4; PGD_2, prostaglandin D_2; TNF, tumour necrosis factor; TARC, thymus activation regulated cytokine (CCL17); TSLP, thymic stromal lymphopoietin.

Couillard S, *et al. Thorax* 2022; 77:933–938. doi: 10.1136/thoraxjnl-2021-217994

APPENDIX 3 – Results of pooled linear mixed effect models exploring the continuous relationships between FeNO and key analytes

Methods

Analytes found to suppress significantly in the primary longitudinal subgroup analyses on FeNO suppressors and non-suppressors (ACQ-5, sputum eosinophils, sputum PGD2, sputum IL-4) were submitted to further analyses exploring their continuous relationship between log-FeNO suppression and analyte-suppression. The 34 patients were analysed in linear mixed effect models with a random intercept for patient to be able to consider each available timepoint, with the independent variable the natural logarithm of FeNO. An unstructured covariance structure was assumed based on AIC and modeling assumptions were all verified visually. Coefficients and their 95% confidence interval are presented to assess the association between significant markers and FeNO suppression.

Results

TABLE E3. Association between selected mediators and ln(FeNO) according to linear mixed models on the entire sample.

Outcome variable	Coefficients	95% C.I.		*p*
ACQ-5	0.89	0.57	1.20	**<0.0001**
Sputum IL-4 (Log-transformed)	0.27	-0.06	0.60	0.1
Sputum eosinophils (Log-transformed)	0.91	0.27	1.55	**0.009**
Sputum PGD2 (Log-transformed)	0.42	0.04	0.80	**0.04**

Bold *p*-values indicate statistical significance ($p<0.05$). ACQ-5, 5-item asthma control questionnaire; C.I., confidence intervals; IL, interleukin; PGD2, prostaglandin D2.

Couillard S, *et al. Thorax* 2022; 77:933–938. doi: 10.1136/thoraxjnl-2021-217994

APPENDIX 4 – Sensitivity analyses for different optimisation methods and test durations

Methods

<u>Summary of different optimisation methods and test durations</u>

An overview of the FeNO suppression test Oxford protocol is provided in Figure E2. Briefly, patients with asthma underwent 7 to 35 days of additional inhaled and/or systemic corticosteroids (+1000µg inhaled fluticasone propionate per day and, if FeNO did not suppress on day 7 according to the equation provided in study methods, +80mg intramuscular (IM) triamcinolone with follow-up 28 days later). Some FeNO suppression tests stopped after 7 days due to patient availability, physician decision, or transition to research bronchoscopy protocols.

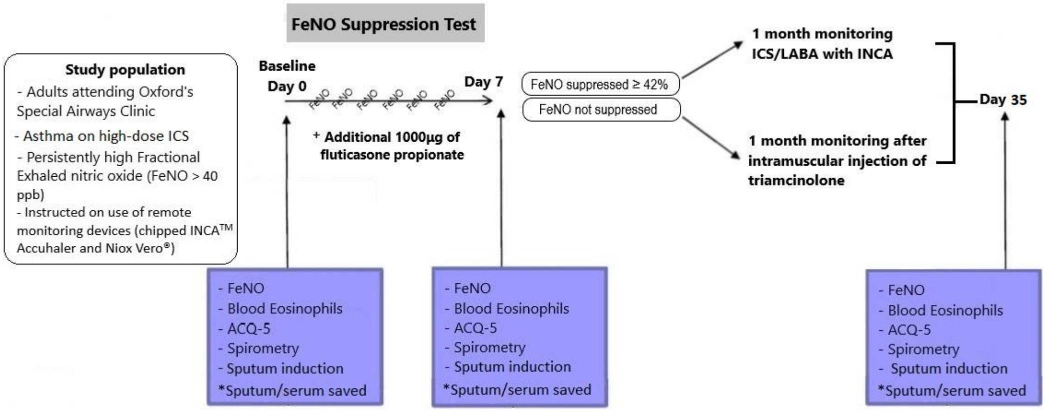

FIGURE E2. The FeNO suppression test, as performed by the Oxford Special Airways Clinic. ACQ-5, 5-item asthma control questionnaire; FeNO, fractional exhaled nitric oxide; GINA, global initiative for asthma; ICS, inhaled corticosteroid; LABA, long-acting beta2-agonist. Figure adapted from Heaney et al. (2019).

Couillard S, *et al. Thorax* 2022; 77:933–938. doi: 10.1136/thoraxjnl-2021-217994

Assessing impact of the optimisation method

Sensitivity analyses of positive findings were conducted to assess whether the final optimisation method for FeNO suppression (*i.e.*: +1000µg inhaled fluticasone propionate or +80mg triamcinolone intramuscularly) resulted in significantly different before/after changes. All study patients were grouped based on the optimisation method. Before *vs* after changes in log_{10}-transformed FeNO and differences in ACQ-5 scores were compared within each group using paired *t*-tests. Fold-changes in FeNO and differences in ACQ (after – before) were compared between the two groups with a Mann-Whitney test and an unpaired t-test, respectively. Furthermore, areas under the curve (AUC) were computed for the log-transformed FeNO values (dependent variable) *vs* time course of FeNO suppression testing (independent variable; segmented in days 0 to 7 and days 7 to 35), with their 95% CI analysed for differences.

Assessing the impact of the test duration

To assess if study duration method (*i.e.* 7- or 35-day test) impacted degree of FeNO suppression and ACQ-5 improvements, all included patients were grouped based on study duration. Before *vs* after changes in log_{10}-transformed FeNO and differences in ACQ-5 scores were compared within each group using paired *t*-tests. Fold-changes in FeNO and differences in ACQ (after – before) were compared between the two groups with a Mann-Whitney test and an unpaired t-test, respectively.

Statistics were analysed with a two-sided α of 0.05.

Couillard S, *et al. Thorax* 2022; 77:933–938. doi: 10.1136/thoraxjnl-2021-217994

Results

Sensitivity analyses on the optimisation method

Patients who did and did not suppress FeNO received triamcinolone 80mg intramuscularly on day 7 in similar proportions (3/15 *vs* 6/19; see main manuscript text, Table 1). The results of the sensitivity analyses are shown in Figure E3 and suggest that, although both optimisation methods were used in different circumstances to ensure optimal FeNO suppression, the magnitude of change did not differ significantly between methods for FeNO (median [IQR], ICS-only vs ICS+IM triamcinolone: ↓2.1[1.4-3.8] *vs* 1.6 [1.3-2.2] -fold, p=0.35) and ACQ (mean±SD: -1.0 ±1.3 *vs* –0.7±0.8, p=0.48). These considerations were further explored in a time course plot (Figure E4), with AUC analyses showing similar degrees of $\log_{10}$-FeNO suppression for both optimisation methods (Table E4).

Couillard S, *et al. Thorax* 2022; 77:933–938. doi: 10.1136/thoraxjnl-2021-217994

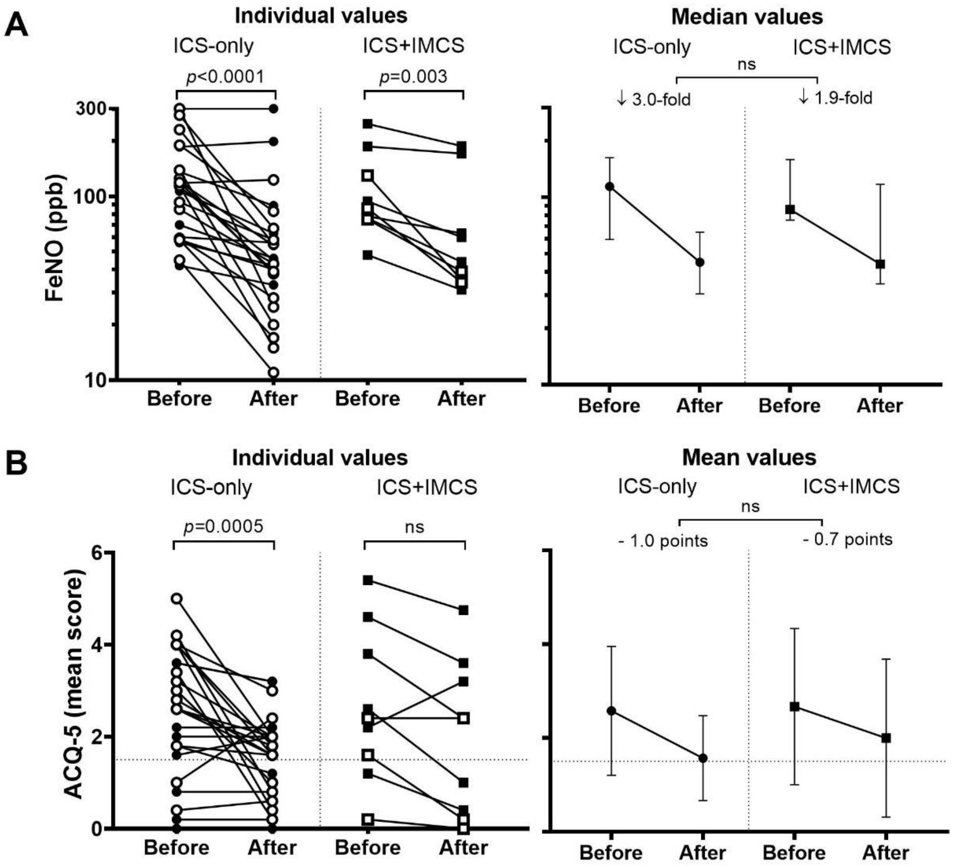

FIGURE E3. Before and after changes in selected analytes following a FeNO suppression test according to the optimisation method. Panel A**:** FeNO, Panel B: 5-item asthma control questionnaire (ACQ-5), with the 1.5-point threshold for good symptom control delimited by the dotted line. ICS, inhaled corticosteroid (*i.e.*: additional fluticasone propionate 1000μg inhaled daily throughout); IMCS, intramuscular corticosteroid (*i.e.*: triamcinolone 80mg intramuscularly on day 7); ○□, FeNO suppressed; ●■, FeNO not suppressed.

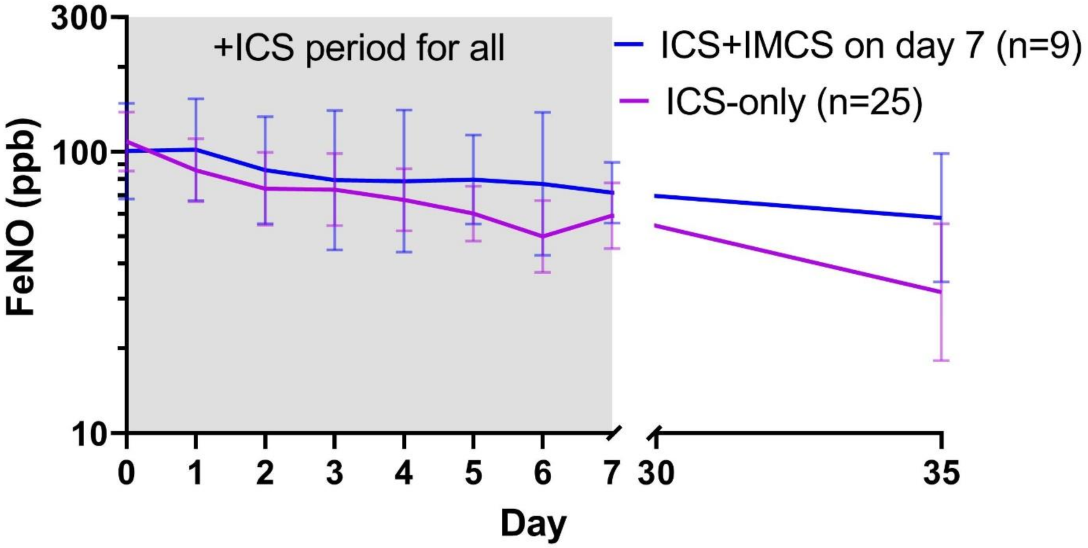

FIGURE E4. FeNO suppression time course according to optimisation method. Full lines connect the geometric mean values at each day of measurement (**bold** day numbers), with error bars corresponding to the 95% CI.

TABLE E4
Area under the curve per segment of the FeNO suppression test according to optimisation method.

**Optimisation method** (n)	**Total area under the curve per segment** **Days 0 to 7**		**Days 7 to 35**		**Days 0 to 35**	
**ICS+IMCS on day 7** (n=9)	13.5	(12.7-14.2)	50.7	(41.7-59.7)	63.0	(54.4-73.9)
**ICS-only** (n=25)	12.9	(11.9-13.9)	45.8	(33.7-58.0)	56.9	(45.6-71.8)
p	ns		ns		ns	

Areas under the curve (95% confidence intervals) shown are computed for mean $\log_{10}$-transformed FeNO values according to time in each subgroup. ICS, inhaled corticosteroid; IMCS, intramuscular corticosteroid; ns, $p \geq 0.05$.

Couillard S, *et al. Thorax* 2022; 77:933–938. doi: 10.1136/thoraxjnl-2021-217994

Sensitivity analyses on the duration of the FeNO suppression test

Patients who did and did not suppress FeNO underwent 7-day tests in similar proportions (5/15 *vs* 11/19; see main manuscript text, Table 1). The results of the sensitivity analyses are shown in Figure E5 and suggest that the magnitude of change did not differ significantly between testing durations for FeNO (median [IQR], 7-day vs 35-day tests: ↓ 1.6[1.3-2.4] *vs* 2.1 [1.5-4.1] -fold, *p*=0.11) and ACQ (mean±SD: -0.6 ±0.9 *vs* -1.2 ± .14, *p*=0.20).

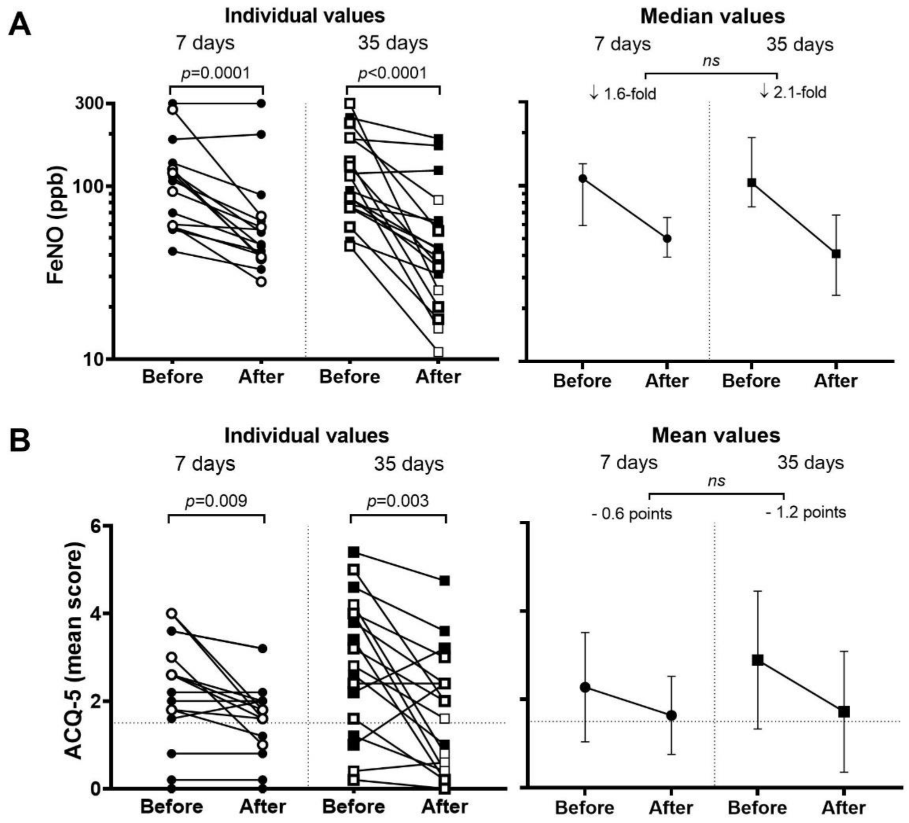

FIGURE E5. Before and after changes in selected analytes following a FeNO suppression test according to duration of test. Panel A**:** FeNO, Panel B: 5-item asthma control questionnaire (ACQ-5), with the 1.5-point threshold for good symptom control delimited by the dotted line. ○□, FeNO suppressed; ●■, FeNO not suppressed.
